# Supplementary material for: Morbidities, health problems, health care seeking and utilization behaviour among elderly residing on urban areas of eastern Nepal: A cross-sectional study
Source: PLoS One. 2022 Sep 7;17(9):e0273101. doi: 10.1371/journal.pone.0273101 (PMC9451091; doi:10.1371/journal.pone.0273101)
Supplement: S1 File — (DOCX) [file pone.0273101.s001.docx]

**A study on unmet health care needs of elderly people residing on urban areas of Sunsari district.**

| **BP KOIRALA INSTITUTE OF HEALTH SCIENCES**  PART A: INTERVIEW |
| --- |
| IDENTIFICATION INFORMATION |
| 1. Respondent ID No. |
| 2. Name of the respondent: |
| 3. Address: House no: Ward no: Street: |
| 4. Date & time of interview: DD/MM/YY |
| 5. Consent has been read and obtained: Yes / No |
| 6. Residential status- 1) Permanent 2) Migrated 3) Rented  8. Type of Family : 1 Nuclear 2 Joint   \| SECTION A: DEMOGRAPHIC PROFILE \| \| --- \| \| 1. Age: 2. Gender: Male / Female \| \| 3. Ethnicity: 4. Religion: \| \| 5. Place of birth: 6. Mother tongue: \| \| 7. Marital status:  1. Never married 2. Currently married 3.Separated  4. Divorced 5. Widowed 6. Cohabiting 7.Refused  8. N/A \| \| 8. Highest level of education completed: _______________  1. No formal schooling 2. Less than primary school 3. Primary school  4. Secondary school 5. SLC/equivalent 6. Intermediate/+2 7.Graduate 8. Post graduate \| \| 9 Work Status:  1.Government employee 2.Non-Government employee  3.Self-employed  4.Non- Paid 5.Student 6.Homemaker  7.Retired   \| 10. Number of family members:  Living Arrangement: 1. with son. 2. With daughter. 3. Couple only. 4 alone. 5. others \| \| --- \| \| 11. Family Income:  Section B : Behavioural Measurements \| \| **A. Smoking**  1. Do you currently smoke any tobacco products, such as cigarettes, cigars, pipes, bidis, hukkahs or tambakhus? 1. Yes 2. No (If no Q 7)  2. Do you currently smoke tobacco products daily? 1. Yes 2. No  3. How old were you when you first started smoking? Or Do you remember how long ago it was? Age (years)………. Don’t know 77  4. On average, how many of the following products do you smoke each day/week?  1. Manufactured cigarettes 2. Hand-rolled cigarettes  3. Pipes full of tobacco  4. Cigars, cheroots, cigarillos 5. Other (please specify): (If less than daily, record weekly)  5. During the past 12 months, have you tried to stop smoking? 1. Yes 2. No  6. During any visit to a doctor or other health worker in the past 12 months, were you advised to quit smoking tobacco?  1.Yes 2. No 3. No visit  7. In the past, did you ever smoke any tobacco products? 1. Yes 2. No  8. In the past, did you ever smoke daily? 1. Yes 2. No  9. How long ago did you stop smoking?  10. Do you currently use any smoke-less tobacco products? 1. Yes 2. No  11 Do you currently use smokeless tobacco products daily? 1. Yes 2. No  12. In the past, did you ever use smokeless tobacco products daily? 1. Yes 2. No  13. During the past 7 days, on how many days did someone in your home/workplace smoke when you were present? …………  **B. Alcohol**  1. Have you ever consumed an alcoholic drink such as beer, wine, Spirits, jaand, rakshi, tongba? 1. Yes 2. NO  2. Have you consumed an alcoholic drink within the past 12 months? 1. Yes 2. NO  3. During the past 12 months, how frequently have you had at least one alcoholic drink?  1. Daily 2. 5-6 days/week 3. 1-4 days/week 4. 1-3 days /month 5. Less than once a month  5. Have you consumed an alcoholic drink within the past 30 days? 1. Yes 2. No  6. During the past 30 days, on how many occasions did you have at least one alcoholic drink? Number: 77. Don’t know  7. During the past 30 days, when you drank alcohol, on average age how many standard alcoholic drinks did you have during one drinking occasion?  Number………………… Don’t know 77  8. During the past 30 days, what was the largest number of standard alcoholic drinks did you have during one drinking occasion? And how many such occasions?  9. Past 30 days how often you consumed with meals?  1. Usually with meals 2. Sometimes 3. Rarely 4. Never  **C. Physical Activity**  1. Does your work involve vigorous intensity activity that causes large increase in breathing or heart rate for at least 10 minutes continuously?  1. Yes 2. No  2. In a typical week on how many days do you do such activities as part of your work? …………  3. How much time do you spend doing vigorous- intensity activities at work on a typical day? …………  4. Does your work involve moderate-intensity activity that causes increase in breathing or heart rate for at least 10 minutes continuously?  1. Yes 2. No  5. If yes how many days as part of your work? …….  6. How much time do you spend doing moderate intensity activities at work on a typical day? ………….  7. Do you walk or use a bicycle (pedal cycle) for at least 10 minutes continuously to get to and from places? 1. Yes 2. No  8. How many days in a typical week? ………  9. How much time do you spend walking or bicycling for travel on a typical day? ………..  10. Sedentary behavior (How much time do you usually spend sitting or reclining on a typical day?) ………….  **D.Diet**   1. In a typical week, on how many days do you eat fruit? Number of days………………. Don't Know 77 2. How many servings of fruit do you eat on one of those days? Number of servings…………… Don't Know 77 3. In a typical week, on how many days do you eat vegetables? Number of days………………. Don't Know 77 4. How many servings of vegetables do you eat on one of those days? Number of servings……………. Don’t know 77 5. How often do you add salt or a salty sauce such as soy sauce to your food right before you eat it or as you are eating it?   1.Always 2Often 3Sometimes 4Rarely 5Never 77Don't know   1. How often is salt, salty seasoning or a salty sauce added in cooking or preparing foods in your household?   Always 1 Often 2 Sometimes 3 Rarely 4 Never 5 Don't know 77   1. How often do you eat processed food high in salt? By processed food high in salt, I mean foods that have been altered from their natural state, such as packaged salty snacks, canned salty food including pickles and preserves, salty food prepared at a fast food restaurant, cheese, bacon and processed meat…Always 1 Often 2 Sometimes 3 Rarely 4 Never 5 Don't know 77 2. How much salt or salty sauce do you think you consume?   Far too much 1 Too much 2 Just the right amount 3 Too little 4 Far too little 5 Don't know 77  **E.History of Raised Blood Pressure**  1. Have you ever had your blood pressure measured by a doctor or other health worker? Yes 1 No 2 if No skip  2. Have you ever been told by a doctor or other health worker that you have raised blood pressure or hypertension? Yes 1 No 2 If no skip  3. Were you first told in the past 12 months? Yes 1 No 2  4. In the past two weeks, have you taken any drugs (medication) for raised blood pressure prescribed by a doctor or other health worker?  Yes 1 No 2  5.Have you ever seen a traditional healer for raised blood pressure or hypertension? Yes 1 No 2  6. Are you currently taking any herbal or traditional remedy for your raised blood pressure? Yes 1 No 2  **F. History of Diabetes**  1.Have you ever had your blood sugar measured by a doctor or other health worker?Yes 1 No 2 If No, skip  2.Have you ever been told by a doctor or other health worker that you have raised blood sugar or diabetes? Yes 1 No 2 If No, skip  3.Were you first told in the past 12 months? Yes 1 No 2  4.In the past two weeks, have you taken any drugs (medication) for diabetes prescribed by a doctor or other health worker? Yes 1 No 2  5.Are you currently taking insulin for diabetes prescribed by a doctor or other health worker? Yes 1 No 2  6.Have you ever seen a traditional healer for diabetes or raised blood sugar? Yes 1 No 2  7.Are you currently taking any herbal or traditional remedy for your diabetes? Yes 1 No 2  **G.History of Raised Total Cholesterol**  1. Have you ever had your cholesterol (fat levels in your blood) measured by a doctor or other health worker? Yes 1 No 2 If No, skip  2. Have you ever been told by a doctor or other health worker that you have raised cholesterol? Yes 1 No 2 If No, skip  3. Were you first told in the past 12 months? Yes 1 No 2  4. In the past two weeks, have you taken any oral treatment for raised total cholesterol prescribed by a doctor or other health worker?  Yes 1 No 2  5. Have you ever seen a traditional healer for raised cholesterol? Yes 1 No 2  6. Are you currently taking any herbal or traditional remedy for your raised cholesterol? Yes 1 No 2  **H. History of Cardiovascular Diseases**  1. Have you ever had a heart attack or chest pain from heart disease (angina) or a stroke (cerebrovascular accident or incident)? Yes1 No2  2. Are you currently taking aspirin regularly to prevent or treat heart disease? Yes 1 No 2  3. Are you currently taking statins regularly to prevent or treat heart disease? Yes 1 No 2  **I. Recreational activities :**  1.Are you involved in any religious activities ? 1 yes 2 No  2.Do you have friends? 1 yes 2 No  3.How much time do you spend with your friends?  4.Are you member in some clubs? 1 Yes 2 No  5.Do you listen to radio/ watch TV regularly? 1 Yes 2 No   \| **Section C : Health and functional abilities** \| \| --- \| \| 1.Are you able to see newspaper print clearly, 1.Yes 2.No and with glasses  if No have you consulted Doctor 1 yes 2 No  2.Are you able to see the face of someone 4m away clearly Y/N and with glasses  3.Are you able to hear distinctly what is said in a conversation with one other person Y/N and with hearing aid  if No have you consulted Doctor 1 yes 2 No  4.Are you able to chew hard foods without difficulty Y/N and using dentures if yes have you consulted  5. Are you diagnosed with any chronic disease? 1 yes 2 No  If yes When …………  6.Are you taking any medications? 1. Yes 2. No  7. Since when are you taking medications? …………..  8. How many medications are you taking? …………..  9. Do you have any past history of chronic illnesses ? if yes specify  10.Do you have a family history of Diabetes Hypertension TB and other chronic disease? …………….  11.Do you have any difficulty doing daily activities like bathing, dressing, toileting, transferring, continence and feeding, as well as instrumental activities of daily living such as food preparation, the ability to use a telephone, housekeeping, shopping and the ability to manage one’s finances? ………  12.Do you have difficulty leaving home without help?................  13. Do you have any urinary problems?  14. Do you have any health problems?  1.Pain / Swelling of Joints 2.Limitation of Movement 3.Indigestion / Heart Bum 4.Backache 5.Excessive Tiredness  /Weakness 6.Breathlessness 7.Headache 8.Cough 9.Giddiness / Fainting 10.Frequency / Urgency 11.Change in Bowel Habits 12.Itching / Infection of Skin 13.Pain / Difficulty in Urination 14.Chest Pain 15.Blurring of vision 16.Pedal edema 17.Wheezing 18.Nasal Congestion 15 19.Others  If yes have you consulted and when  **PART D:  Use of health care resources**  1. Where do you usually go to when you are sick?  1. hospital 2. clinic 3.doctor’s private practice 4.traditional healer/prophet  3. What is the reason for your choice in question 2?  4. How do you get there?  1. by walking  2. by taxi  3. by bus  4. own vehicle  5. How long does it take you to get to there? 1. <30mins 2.>30 mins  6. Do you have a regular doctor that you see for your disease? Yes… No…  11When was the last time you visited hospital and for what cause?  1. 6months 2. 1 year 3. 2 year  2.How far is the nearest health facility from your home?  1. ≤30 mins 2. >30mins  3. Who brought to the hospital then?  1. Yourself 2. Parents 3. Friends 4. Wife 5. Children  4. How many times did you visit the hospital/clinic in the last 6 months? ………  5. How many times did you visit the private doctor in the last 6 months? ………  6. How many times did you visit an emergency room in the last 6 months? ……  7. How many times did you have to stay overnight in the hospital in the last 6 months ?  PART E:  Factors for Unmet health care needs  1.Why didn’t you visited hospital for your problems?  1Low availability 2high cost 3 unable to walk(activity restriction)  2. Do you have difficulty visiting health care: 1. Yes. 2. No  **Part F :Knowledge regarding health:**   1. About free medicines available in policy : 1. Yes 2.No 2. About free health treatments available : 1. Yes 2. No 3. About priority given in health institution for elderly : 1. Yes 2. No   **Section G : Physical measurements**  Blood Pressure  Reading 1  Reading 2  Reading 3  During the past two weeks, have you been treated for raised blood pressure with drugs (medication) prescribed by a doctor or other health worker? Yes 1 No 2  Height and Weight  Height in Centimetres (cm)  Weight in Kilograms (kg)  Waist in centimetres (cm)  Hip Circumference and Heart Rate  Hip circumference in Centimeters (cm)  Heart Rate Reading 1 Beats per minute Reading 2 Beats per minute Reading 3 Beats per minute  Dipstick urine measurement: Protein sugar Ph  **Beck Depression Inventory 2nd edition (BDI-II)**  **Sadness**  0. I do not feel sad.  1. I feel sad much of the time.  2. I am sad all the time.  3. I am so sad or unhappy that I can’t stand it.  **Pessimism**  0. I am not discouraged about my future.  1. I feel more discouraged about my future than I used to be.  2. I do not expect things to work out for me.  3. I feel my future is hopeless and will only get worse.  **Past Failure**  0. I do not feel like a failure.  1. I have failed more than I should have.  2. As I look back I see a lot of failures.  3. I feel I am a total failure as a person.  **Loss of Pleasure**  0. I get as much pleasure as I ever did from the things I enjoy.  1. I don’t enjoy things as much as I used to.  2. I get very little pleasure from the things I used to enjoy.  3. I can’t get any pleasure from the things I used to enjoy.  **Guilty Feelings**  0. I don’t feel particularly guilty.  1. I feel guilty over many things I have done or should have done. 115  2. I feel guilty most of the time.  3. I feel guilty all the time.  **Punishment Feelings**  0. I don’t feel I am being punished.  1. I feel I may be punished.  2. I expect to be punished.  3. I feel I am being punished.  **Self-Dislike**  0. I feel the same about myself as ever.  1. I have lost confidence in myself.  2. I am disappointed in myself.  3. I dislike myself.  **Self-Criticalness**  0. I don’t criticize or blame myself more than usual.  1. I am more critical of myself than I used to be.  2. I criticize myself for all of my faults.  3. I blame myself for everything bad that happens.  **Suicidal Thoughts or Wishes**  0. I don’t have any thoughts of killing myself.  1. I have thoughts of killing myself, but I would not carry them out.  2. I would like to kill myself.  3. I would kill myself if I had the chance.  **Crying**  0. I don’t cry any more than I used to.  1. I cry more than I used to.  2. I cry over every little thing.  3. I feel like crying, but I can’t.  **Agitation**  0. I am no more restless or wound up than usual.  1. I feel more restless or wound up than usual.  2. I am so restless or agitated that it’s hard to stay still.  3. I am so restless or agitated that I have to keep moving or doing something.  **Loss of Interest**  0. I have not lost interest in other people or activities.  1. I am less interested in other people or things than before.  2. I have lost most of my interest in other people or things.  3. it’s hard to get interested in anything.  **Indecisiveness**  0. I make decisions about as well as ever.  1. I find it is more difficult to make decisions than usual.  2. I have much greater difficulty in making decisions than I used to.  3. I have trouble making any decisions.  **Worthlessness**  0. I do not feel I am worthless.  1. I don’t consider myself as worthwhile and useful as I used to.  2. I feel more worthless as compare to other people.  3. I feel utterly worthless.  **Loss of Energy**  0. I have as much energy as ever.  1. I have less energy than I used to have.  2. I don’t have enough energy to do very much.  3. I don’t have enough energy to do anything.  **Changes in Sleeping Pattern**  0. I have not experienced any change in my sleeping pattern.  1. I sleep somewhat less than usual. –or–  I sleep somewhat more than usual.  2. I sleep a lot less than usual. –or–  I sleep a lot more than usual.  3. I sleep most of the day. –or–  I wake up 1-2 hours early and can’t get back to sleep. Annexures  **Irritability**  0. I am no more irritable than usual.  1. I am more irritable than usual.  2. I am much more irritable than usual.  3. I am irritable all the time.  **Changes in Appetite**  0. I have not experienced any change in my appetite.  1. My appetite is somewhat less than usual. –or–  My appetite is somewhat greater than usual.  2. My appetite is much less than usual. –or–  My appetite is much greater than usual.  3. I have no appetite at all. –or–  I crave food all the time.  **Concentration Difficulty**  0. I can concentrate as well as ever.  1. I can’t concentrate as well as usual.  2. It’s hard to keep my mind on anything for very long.  3. I find I can’t concentrate on anything.  **Tiredness or Fatigue**  0. I am no more tired or fatigued than usual.  1. I get more tired or fatigued more easily than usual.  2. I am too tired or fatigued to do a lot of the things I used to do.  3. I am too tired or fatigued to do most of the things I used to do.  **Loss of Interest in Sex**  0. I have not noticed any recent change in my interest in sex.  1. I am less interested in sex than I used to be.  2. I am much less interested in sex now.  3. I have lost interest in sex completely.  Client Name ______________________________ Date: ____________________ Subtotal Page 1 __________ Subtotal Page 2 __________ Total Score __________ Score of 0-13: minimal, 14-19: mild, 20-28: moderate, and 29-63: severe    In local Language: (Font used: Preeti)  v08–sM ;fdflhs hg;ª\lVos ljj/0fM  != y/÷hflt ===================================================== @= pd]/================#= lnª==========$=sIffM s_ !! v_ !@ ^= wd{M======================== &= ;Dks{ gDa/M=================================  z}lIfs of]UotfM s_ c;fIf/ v_ ;fIf/  olb ;fIf/ ePdf s_ cf}krfl/s lzIff -plTt0f{ dflyNnf] tx_============================== v_ cgf}krfl/s lZfIff  &= kl/jf/df slt hgf ;b:o x'g'x'G5 <=========  **v08–vM Jojxfl/s ljj/0f**  **c= ;'lt{ ;]jgsf] ljj/0fM**  != s] tkfO{n] slxNo} w'jf kmfNg] ;'lt{hGokbfy{ h:t} r'/f]6, l;uf/, lrnLd, lj8L, x'Ssf, tdfv' cflb ;]jg ug'{ ePsf] 5 < s_ 5 v_ 5}g  @= tkfO{n] slt jif{sf] pd]/df ;'lt{hGo kbfy{ ;]jg ug{ yfNg'eof] <=======================  #= tkfO{n] klxnf] k6s s;sf] ;fydf ;'lt{hGo kbfy{ ;]jg ug'{eof] <  s_ ;fyL v_ gft]bf/-pNn]v ug'{xf];_===================================================== u_ PSn}  $= s] tkfO{ xfn dfly pNn]v kbfy{ ;]jg ug'{x'G5 < s_ u5'{ v_ ulb{g  %= s] tkfO{ b}lgs ?kdf ;'lt{hGo kbfy{ ;]jg ug'{x'G5 < s_ u5'{ v_ ulb{g  ^= cf};tdf lgDg pNn]lvt ;'lt{hGo kbfy{x? ! lbgdf sltj6f;]jg ug'{xG5 <  s_ r'/f]6========== v_ lj8L=========== u_ tDjfv'============ 3_ l;uf/========ª_ cGo======  &= ljut !@ dlxgfdf, s] tkfO{n] w'd\|kfg 5f8\g] k\|of; ug'{ePsf] 5 < s_ 5 v_ 5}g  (= ljutdf s] tkfO{n] slxNo} w'd\|kfg ug\e'{ of] < s_ u/] v_ ul/g  !)= ljutdf s] tkfO{n] lbgx' wd\|kfg ug'{eof] < s_ u/] v_ ul/g  !!= tkfO{n] w'd\|kfg ug{ slt cufl8 5f8\g' eof] < ================= lbg÷xKtf÷dlxgf÷jif{ cufl8  !@= xfn s] tkfO{ w'jf gkmfNg] ;'tL{hGo kbfy{ -v}gL, ;'tL{, u'6vf, cflb_ ;]jg ug'{x'G5< s_ u5'{ v_ ulb{g  !#= s] tkfO{ w'jf gkmfNg] ;'tL{hGo kbfy{ xfn lbgx' ;]jg ug'{x'G5 < s_ u5'{ v_ ulb{g  !$= s] tkfO{n] ljutdf slxNo} w'jf gkmfNg] ;'tL{hGo kbfy{ ;]jg ug'{ x'GYof] < s_ uy]{ v_ ulb{gy]  !%= ljutsf] ;ft lbgdf slt lbg h;f] s;}n] -;fyL, cfkmGt cflb_ n] tkfO{ ;+u} ePsf] j]nf w'd\|kfg ug'{eof] < ============ lbg  **cfM dfbs kbf{y ;]jgsf] ljj/0fM**  != s] tkfO{n] slxNo} dfbs kbf{y -h:t} hfF8, /S;L, ljo/, tf]ªjf, jfOg cflb_ ;]jg ug'{ ePsf] 5 <  s_ 5 v_ 5}g  @= tkfO{n] klxnf] k6s s;sf] ;fydf dfbs kbfy{ ;]jg ug'{eof] <  s_ ;fyL v_ gft]bf/-pNn]v ug'{xf];_=============================================== u_ PSn }  #= s] tkfO{n] ljut !@ dlxgfdf slxNo} dfbs kbfy{ ;]jg ug'{ePsf] 5 < s_ 5 v_ 5}g  $= ljutsf !@ dlxgfdf slt k6s h:tf] tkfO{n] slDtdf Ps dfbs kbfy{ (one drink) ;]jg ug'{ ePsf] 5 <  s_ b}lgs v_ xKtfdf %–^ lbg u_ xKtfdf !–$ lbg 3_ dlxgfdf !–# lbg ª_ dlxgfdf ! k6s eGbf sd  %= ljutsf #) lbgdf s] tkfO{n] slxNo} dfbs kbfy{ ;]jg ug'{ePsf] 5 < s_ 5 v_ 5}g  ^= ljutsf #) lbgdf tkfO{n] slt pknIodf slDtdf Ps dfbs kbfy{ (one drink) ;]jg ug'{eof] <  s_ =========== k6s v_ yfxf 5}g  &= tkfO{n] clGtd k6sdf s'g s'g dfbs kbfy{ -h:t} Xljl:s, ef]8\sf, /d, ljo/ cflb_ / slt dfqfdf -h:tM}  slt k]u, ldln, af]6n_ ;]jg ug'{ePsf] lyof] <================================= ===============  **OM vfgfsf] ljj/0f**  != tkfO{ ! xKtfdf slt lbg h;f] kmnk'mn ;]jg ug'{xG5 < ==============lbg  @= oL lbgx? dWo]sf Pslbgdf tkfO{ s'g s'g kmnkm'n -:ofp, ;'Gtnf, s]/f cflb_ slt dfqfdf -l;ª\u},sltj6f,  rfgf, cflb_ lng'x'G5 <========================== ======= ==================================== ==========  #= tkfO{ xKtfdf slt lbg h;f] ;fu;AhL ;]jg ug'{xG5 < ===========lbg  $= oL lbgx? dWo]sf Pslbge/Ldf tkfO{ slt dfqfdf -slt srf}/f_ ;fu;AhL lng'x'G5 < ===============srf}/f  %= tkfO{sf] 3/df vfgf ksfpbf s'g k\|sf/sf] t]n÷l3p k\|of]u ug'{x'G5 <  s_+ tf]/Lsf] t]n v_ k\|;f]lwt ag:klt t]n u_ ;'o{d'vL t]n 3_ l3p÷dVvg ª_ cGo==============  ^= cf};tdf tkfO{ Ps xKtfdf slt k6s 3/df gksfPsf] vfg]s'/f -vfhf, vfgf_ aflx/ xf]6n, /]i6'/]06, 7]nf cflbdf  vfg'x'G5 < ================  **O{M g"gsf] ljj/0f**  != vfgf vfg' cufl8 jf vfb} ubf{ tkfO{ sltsf] g"g yKg' x'G5 <  s_ ;w} v_w]/} h;f] u_ slxn] sflx 3_ lj/n} ª_ slxNo} klg ulb{g  @= g"g a9L ePsf] tof/L vfg]s'/f-h:t}M rfprfp, n]h, s'/s'/], g'gLnf] lj:s'6, lttf}/f, Kofs]6sf] crf/ cflb_  sltsf] vfg'x'G5 <  s_ ;w} v_ w]/} h;f] u_ slxn] sflx 3_ lj/n} ª_ slxNo} klg vflbg  #= tkfO{ g"g slt dfqfdf vfg'x'G5 h:tf] nfU5 <  s_ w]/} g} a9L v_ a9L u_ l7s dfqf 3_ sd  ª_ w]/} g} sd r_ yfxf 5}g  $= s] tkfO{nfO{ nfU5, tkfO{sf] vfgfdf g"gsf] a9L dfqfn] uDeL/ :jf:Yo ;d:of x'g;S5 <  s_ nfU5 v_ nfUb}g u_ yfxf ePg  ^= tkfO{ s'gk\|sf/sf] g"g k\|of]u ug'{x'G5 <  s_ l9Ss] g"g v_ lrGx lagfsf] w'nf] g"g  u_ cfof] g'g -b'O{ afnaflnsfsf] lrGx ePsf] w'nf] g"g_  **pM zf/Ll/s lqmofsnfksf] ljj/0f**  **e\|d0fsf] ljj/0f**  != s] tkfO{ Ps 7fpFb]lv csf]{ 7fpF hfg lg/Gt/ !) ldg]6 ;Dd lx8\g' jf ;fOsnsf] k\|of]u ug'{x'G5 <  s_ u5'{ v_ ulb{g  @= xKtfdf tkfO{ slt lbg !) ldg]6;Dd lg/Gt/ lx8\g' jf ;fOsnsf] k\|of]u ug'{x'G5 < ===============lbg  #= b}lgs ?kdf tkfO{ slt ;do lx8\g] jf ;fOsn rnfP/ vr{g' x'G5 <================= ldg]6÷306f  $= s] tkfO{ s'g} hf]8\bf/ lta\|tfsf] v]bns'b, tGb'?l:t jf dgf]/~hgsf lqmofsnfk ug'{x'G5 h;n] tkfO{sf] Zjf;  jf d'6'sf] w8\sg slDtdf klg !) ldg]6;Dd lg/Gt/ a9\5 < -h:t} bf}8g], km'6an v]Ng] cflb_  s_ u5'{ v_ ulb{g  %= dfly pNMn]lvt lqmofsnfkx? xKtfdf slt lbg ug'{x'G5 <============ lbg  ^= Ps lbgdf dfly pNn]lvt lqmofsnfkx? slt ;do;Dd ug'{x'G5 < ================ ldg]6  &= s] tkfO{ dWo lta\|tfsf] v]ns\'b, tGb'?l:t jf dgf]/~hgsf lqmofsnfkx?-h:t} lx88'n, ;fOsn s'[bfpg], kf}l8  v]Ng], elnjn, cflb_ ug'{x'G5 h;n] tkfO{sf] :jf; jf d'6'sf] w8\sg lg/Gt/ slDtdf !) ldg]6 ;Dd s]xL a9fpg]  ub{5 < s_ u5'{ v_ ulb{g  *= dfly pNMn]lvt lqmofsnfkx? xKtfdf slt lbg ug'{x'G5 < ============ lbg  (= Ps lbgdf dfly pNn]lvt lqmofsnfkx? slt ;do;Dd ug'{x'G5 < ============ ldg]6  !)= tkfO{ Ps lbgdf slt ;do h:tf] j;]/÷9Ns]/÷l6eL x]/]/ jf OG6/g]6÷km];a's÷Rofl6ª÷lel8of] Uf]d cflb  rnfP/ ljtfpg'x'G5 <===========ldg]6÷306f  !!= tkfO{ /ftdf slt ;do h:tf] ;'Tg'x'G5 < =============== 306f  **v08 uM ljutsf] ljj/0f**  **cM pRr /Strfksf] ljj/0f**  != s] tkfO{n] slxNo} cfkm\gf] /Strfk 8fS6/ jf cGo :jf:YosdL{nfO{ hrfpFg'ePsf] 5 <  s_ 5 v_ 5}g  @= s] tkfO{nfO{ 8fS6/ jf cGo :jf:YosdL{n] tkfO{sf] /Strfk a9]sf] jf pRr /Strfk ePsf] eGg' ePsf] 5 <  s_ 5 v_ 5}g  olb 5 eg] slxn]b]lv tkfO{nfO{ pRr /Strfk ePsf] elgPsf] xf] <==============dlxgf÷jif{ cufl8b]lv  #= s] tkfO{n] xfndf pRr /Strfksf] nflu cf}ifwL ;]jg u/L /xg'ePsf] 5 < s_ 5 v_ 5}g  **cfM dw'd]xsf] ljj/0f**  != s] tkfO{n] slxNo} cfkm\gf] /utdf lrgLsf] dfqf 8fS6/ jf cGo :jf:YosdL{af6 k/LIf0f u/fpg' ePsf] 5 <  s_ 5 v_ 5}g  @= s] tkfO{nfO{ 8fS6/ jf cGo :jf:YosdL{n] tkfO{sf] /utdf lrgLsf] dfqf a9]sf] cyjf dw'd]x (diabetes) ePsf]  eGg' ePsf] 5 < s_ 5 v_ 5}g  olb 5 eg] slxn]b]lv tkfO{nfO{ dw'd]x ePsf] elgPsf] xf] <=============dlxgf÷jif{ cufl8b]lv  #= s] tkfO{n] xfndf dw'd]xsf] nflu cf}ifwL ;]jg u/L /xg'ePsf] 5 < s_ 5 v_ 5}g  **sf]n]Z6/f]nsf] laj/0f**  ! s] tkfO{n] slxNo} cfkm\gf] /utdf af];f]sf] dfqf k/LIf0f u/fpg' ePsf] 5 < s_ 5 v_ 5}g  @ s] tkfO{nfO{ 8fS6/ jf cGo :jf:YosdL{n] tkfO{sf] /utdf af];f] dfqf a9]sf] eGg' ePsf] 5 < s_ 5 v_ 5}g  # s] tkfO{n] xfndf dw'd]xsf] nflu cf}ifwL ;]jg u/L /xg'ePsf] 5 < s_ 5 v_ 5}g  **d'6' /f]u ljj/0f**  ! tkfO{nfO{ d'6' b'Vg], d'6' /f]u, :6\|f]s< s_ 5 v_ 5}g  @ s] tkfO{n] xfndf d'6' /f]usf nflu (aspirin) cf}ifwL ;]jg u/L /xg'ePsf] 5 < s_ 5 v_ 5}g  # s] tkfO{n] xfndf d'6' /f]usf nflu (statin) cf}ifwL ;]jg u/L /xg'ePsf] 5 < s_ 5 v_ 5}g  **;fdfhLs lqmofsnfk**  ! wld{s sfo{x?df slQsf] ?rL /fVg'x'G5 <  @ ;fyLx?;Fu slQsf] ;do latfpg'x'G5 <  # s'g} ;F3 ;+:yfdf cfa4 x'g'x'G5 <  **:jf:Yo / lqmofTds ;Ifdtf**  ! klqsfdf n]lvPsf] cIf/ :ki6 b]Vg ;Sg'x'G5 <  @ $ ld6/ k/ af6 dfG5]sf] cg'xf/ lrGGf'x'G5 <  # s'/fsfgL ubf{ c?n] af]n]sf] k\|i6 ;'Gg'x'G5 <  $ s'g} cK7]/f] lagf ;fx\|f] vfgf rafpg ;Sg'x'G5 <  % tkfO{nfO{ s'g} bL3{ /f]u 5 <  ^ s'g} cf}ifwL ;]jg ub}{ x'g'x'G5 <  & tkfOnfO klxnf s'g} /f]u nfu]sf] lyof] <  * b}lgs lqmofsnfksf] nfuL tkfOnfO{ slQsf] cK7]/f] 5 <  ( s;}sf] ;xfotf lagf 3/af6 aflx/ hfg ;Sg'x'G5 <  !) tkfOnfO clxn] :jf:Yodf s'g} ;d:of 5 < olb 5 eg] lrlsT;s;Fu e]6 ug'{ eof] <  !! tkfOnfO{ cfkm\gf] :jf:Yo s:tf] eP h:tf] nfU5 < /fd\|f] l7s} g/fd\|f]  **:jf:Yo ;\|f]tsf] pkof]u**  ! la/fdL xF'bf sxFf hfg'x'G5 < !xl:k6n @kmfd]{;L #k\|fOe]6 8fS6/ $wfdL / lsg  @ s;/L hfg'x'G5 < !lx8\b} @6\ofS;L # a; $cfkm\gf} ;fwg  # slt ;do nfU5< ! < #)dL. @ > #)dL  $ tkfO{ cfkm\gf] :jf:Yo ;d:ofsf] nfuL wfdLsf]df slQsf] hfg'x'G5 < ;Fw} k\|fo slxn]sfxLF slxNo} hfFlbg  % clGtd k6s c:ktfn slxn] hfg'eof] < ^ dlxgf ! aif{ # aif{  ^ uPsf] ^ dlxgfdf slt k6s c:ktfn hfg'eof] <  & uPsf] ^ dlxgfdf O{d/h]G;L hfg'kof]{ <  * uPsf] ^ dlxgfdf s'g} sf/0fn] c:ktfndf egf{ x'g'eof] <  ( :jf:Yo ljdf u/fpg' ePsf] 5 <  !) :jf:Yo ;]jf;Fu tkfO{ slQsf] ;Gt'i6 x'g'x'G5 <  **gk'u]sf] :jf:Yo dfu**  tkfO{ cfkm\gf] ;d:ofsf] nfuL :jf:Yo ;]jf lng lsg hfg'ePg <  tkfO{nfO{ c:ktfn hfg s]lx ;d:of 5 < 5 eg] s:tf ;d:of  tkfOnfO{ a[4nfO{ lbPsf] ;]jf P]g af/] yfxf 5 <  v08 ªM zf/Ll/s dfkg  != prfOM ===========;]=ld= @= tf}nM ===============s]=hL=  #= sDd/sf] (waist) kl/lwM ==========;]=ld $= lxksf] kl/lwM ===========;]=ld  %= /Strfk (Blood Pressure) ^= gf8Lsf] rfn (Pulse Rate)  !_ ========================== mmHg ============================/min  @_ ========================== mmHg ============================/min #_ ========================== mmHg ============================/min  ;fwf/0f kl/If0fM  lk;fasf] kl/If0f M k\|f]6Lg Un'sf]h  **a]s l8K/]zg O{Ge]g6f]/L @**  gf]6, s[kof tnsf k\|Zgx?sf ;fy} pQ/ klg k9\g' xf]nf .  s[kof lt nIf0fx?n] tkfO{nfO{ uPsf] @ xKtfdf slQsf] lk/Nof] /fd\|/L ;f]r]/ hjfkm lbg' xf]nf .  !. uPsf] b'O{ xKtfdf tkfO{ slQsf] pbf; x'g'x'GYof] <  slxn] eO{g ==================================================================================)  k\|fo ePF=======================================================================================!  ;w}+ h;f] ePF ==============================================================================@  c;x\o ePsf] lyof] =======================================================================#  @. uPsf] b'O{ xKtfdf tkfO{ slQsf] lg/fz x'g' eof] < -h:t} cfˆgf] eljiosf] af/]df ;Dem]/_  slxn] eO{g ==================================================================================)  k\|fo ePF=======================================================================================!  ;w}+ h;f] ePF ==============================================================================@  c;x\o ePsf] lyof] =======================================================================#  #. uPsf] b'O{ xKtfdf tkfO{nfO{ cfkm\gf] lhjgdf slQsf] c;kmn ePh:tf] nfUof]<  slxn] nfu]g ===================================================================================)  k\|fo nfUof] ======================================================================================!  ;w}+ h;f] nfUof] ===============================================================================@  c;x\o eof]/ k"0f{?kn] c;kmn eP h:tf] nfUof] =======================================#  $. uPsf] b'O{ xKtfdf tkfO{ cfkm\gf] lhjg b]vL slQsf] c;Gt'i6 x'g'eof] <  slxn] c;Gt'i6 eO{g ======================================================================)  k\|fo c;Gt'i6 ePF============================================================================!  ;w}+ h;f] c;Gt'i6 ePF ===================================================================@  lhjgsf] x/]s s'/fdf c;Gt'i6 ePF ====' ==============================================#  %. uPsf] b'O{ xKtfdf tkfO{n] cfkm\gf] lhGbuLsf] nflu cfkm'nfO{ slQsf] bf]lif 7fGg'eof] <  slxn] bf]lif 7flgg==========================================================================)  k\|fo bf]lif 7fg]+ ============================================================================!  ;w}+ h;f] bf]lif 7fg]+ =====================================================================@  lhjgsf] x/]s s'/fdf bf]lif 7fg]+ =====================================================#  ^. uPsf] b'O{ xKtfdf tkfO{n] cfkm\gf] lhGbuLdf slQsf] b'v of ;fl:t ef]Ug' eof] <  slxn] ;fl:t ef]lug=================================================================)  k\|fo ;fl:t ef]u]+ ===================================================================!  ;w}+ h;f] ;fl:t ef]u]+==============================================================@  lhjgsf] x/]s s'/fdf ;fl:t ef]u]+===============================================#  &. uPsf] b'O{ xKtfdf tkfO{nfO{, cfkm'b]vL cfkm'nfO{ slQsf] jfSs nfUof] <  slxn] jfSs nfu]g ==================================================================)  w]/}h;f] jfSs nfUof] =======================================================================!  ;w}+ jfSs nfUof] ============================================================================@  cfkm'nfO{ g}+ 3[0ff nfUof]=====================================================================#  *. uPsf] b'O{ xKtfdf tkfO{ cfkm\gf] ulNtx? k\|lt slQsf] lhDd]jf/ x'g'\eof]<  lhDd]jf/ eO{g =================================================================================)  ulNt / sdhf]/Lsf] nflu pQ/bfoL ePF= ==============================================!  sdhf]/Lx?sf] lgGbf u/]sf] lyPF=============================================================@  x/]s g/fd\|f s'/fk\|lt lhDd]jf/ ePF=== ====================================================#  (. uPsf] b'O{ xKtfdf tkfO{n] cfkm'nfO{ slQsf] xfgL k'Øfpg rfxg' eof] <  cfkm'nfO{ s'g} klg xfgL k'Øfpg rfxLg ===============================================)  xfgL k'Øfpg rfx]+ t/ s]xL ug{ ;sLg == =============================================!  d cfkm'nfO{ g} dfg{ rfxGy]+==================================================================@  d}n] df}sf kfpg] lalQs} cfTdxTof uy{]=+==================================================#  !). uPsf] b'O{ xKtfdf slQsf] ?g' eof] <  klxn] eGbf a9L /f]Og =======================================================================)  klxn] eGbf a9L /f]PF =========================================================================!  xKt} e/L /f]PF==================================================================================@  rfx]/ klg ?g ;lsg+==========================================================================#  !!. uPsf] b'O{ xKtfdf tkfO{nfO{ slQsf] 56\k6L eof] <  klxn] eGbf sd nfUof]======= ====== =======================================================)  klxn] eGbf a9L nfUof] ====== == ============= ================================================!  ;fx\|} 56\k6L nfUof]==================================================================================@  ;fx\|} 56\k6L eP/ s]lx ug{ ;s]g==================================================================#  !@. uPsf] b'O{ xKtfdf tkfO{nfO{ c?;Fu slQsf] xfF;v]n ug{ dg nfUof] <  xfF;v]n ug{ dg nfUof] ===============================================)  klxn] h:tf] xfF;v]n ug{ dg nfu]=============================!  af]Ng klg dg nfu]g ===================================================@  s;}sf] s'g} jf:tf /fVg dg nfu]g==================================#  !#. uPsf] b'O{ xKtfdf tkfO{n] cfkm\gf] lhjgdf slQsf] lg0f{o lng ;Sg'eof] <  lg0f{o lng ;Sg'eof=====================================================)  klxn] h:t} lg0f{o lng ;sLg =========================================!  lg0f{o lng lgs} ufXf] eof]=================================================@  s'g} klg lg0f{o lng ;sLg=============================================#  !$. uPsf] b'O{ xKtfdf tkfO{n] cfkm'nfO{ slQsf] dxTjk"0f{ 7fGg' x'G5 ?  klxn] hlQs} 7fg]======= =================================================)  klxn] hlQs} 7flgg ====== == ============= ==================================!  c/m eGbf sd 7fg] ============================================@  k6Ss} 7flGbg==========================================================#  !%. uPsf] b'O{ xKtfdf tkfO{n} slQsf] sfd ug{ ;Sg'eof] <  klxn] hlQs} sfd ug{ ;s]+ ===================================)  klxn] h:tf] sfd ug{ ;sLg ======= ================================!  sfd ug{ Psbd ufXf] eof] ============================================@  s]xL klg sfd ug{ ;sLg =====================================================#  !^. uPsf] b'O{ xKtfdf tkfO{ slQsf] ;'Tg' eof] <  klxn] hlQs} ;'t]+ ====== ==============================================)  klxn] h:tf] ;'Tg ;sLg================== ==============================!  klxn] eGbf cuf8L lapFlemGy]+ / lgbfpg ;sLg ====================@  Psbd} rfF9} lapFlemGy]+ / lgbfOg=============================================#  !&. uPsf] b'O{ xKtfdf tkfO{nfO{ slQsf] emsf]{ nfUof]<  slxn] eO{g ==============================================================)  k\|fo ePF=======================================================================!  ;w}+ h;f] ePF ==================================================================@  c;x\o ePsf] lyof] ============================================================#  !*. uPsf] b'O{ xKtfdf tkfO{nfO{ vfgf slQsf] ?rLeof] <  klxn] hlQs} ?rL eof] ======= =================================)  klxn] hlt ?rL ePg ==============================================!  Psbd} sd ?rL eof] ============ ==================================@  k6Ss} ?rL ePg==========================================================#  !(. uPsf] b'O{ xKtfdf tkfO{nfO{ slQsf] sfddf Wofg nfUof] <  klxn] hlQs} Wofg nfUof] ======= ====== =========================================)  klxn] hlQs} Wofg nfu]g====== == ============= =====================================!  s'g} klg sfddf nfdf] ;do;Dd Wofg lb]g ;s]g======================@  s'g} klg sfddf Wofg lb]g ;s]g ===================================================#  @). uPsf] b'O{ xKtfdf tkfO{ slQsf] yfSg' eof] <  yfSg' ePg]+ ================== =================================)  klxnf eGbf a9L yfs]+=========== ===============================!  h] ubf{ klg yfSby]+ ================================================@  Psbd} yfs]/ s]lx klg ug{ ;sLg ==============================#  @!. uPsf] b'O{ xKtfdf tkfO{n] cfkm\gf] lhjg ;fyLsf] af/]df slQsf] ;f]Rg' eof] <  klxnf hlQs} ;f]r]+ ============== =============================)  klxnf hlQs} ;f]rLg=============================================!  Tolt jf:tf g}+ ePg ==================== =========================@  jf:tf g}+ ePg ========================== =============================# \| \| \| |
