# Supplementary material for: Morbidities, health problems, health care seeking and utilization behaviour among elderly residing on urban areas of eastern Nepal: A cross-sectional study
Source: PLoS One. 2022 Sep 7;17(9):e0273101. doi: 10.1371/journal.pone.0273101 (PMC9451091; doi:10.1371/journal.pone.0273101)
Supplement: Attachment — Submitted filename: Response to reviewers.docx [file pone.0273101.s003.docx]

Reviewer 1:

Thank you for the constructive comments.

**R1 Comment 1**: Line 57: What are the challenges and additional demands?

Response: The challenges are to respond to the increasing health care demand and needs of elderly with increasing age. This line now reads as, “Morbidity increases with age, thus health care systems need to continually update in order to adequately cater for these additional demands.” 55-56.

**R1 Comment 2:** Lines 60-63: It is not clear what the unmet needs were and the purpose/objective of follow-up.

Response: The unmet needs are defined as no health care consultation despite of persistent health needs. I have edited this line and it now reads as “Lower survival probabilities for individuals with unmet health care needs are reported. Moreover, unmet health needs conferred a higher risk of mortality, with excess risk ranging from 10% to 155%” in line 58 - 60.

**R1 Comment 3:** Line 96: Numbers less than ten should be spelled out in academic writing Line

Response: This has been edited and the numbers are written in text accordingly.

**R1 Comment 4:** 122: Check the spelling of “than” or “then”. “the complaints and findings were then….”

Response: This has been edited in line 140.

**R1 Comment 5:** Line 166: Check spellings

Response: We have checked all the spellings accordingly.

**R1 Comment 6:** Lines 165-169: Logistic regression is missing in the result section. Proteinuria as a variable is not mentioned in the methods section.

Response: We have added new tables with regression. Proteinuria is removed from methods as we are not reporting it in results.

**R1 Comment 7:** Line 236: The term “developing country” may not be appropriate. The authors may consider using another term and also instead of using “like ours”, it is preferable to use “like/such as Nepal”.

Response: We have edited the text accordingly and rephrased the paragraph.

**R1 Comment 8:** Lines 235-239: The first paragraph of the discussion should mention the major findings of the study. Discussion: Do not repeat sentences from the result section in discussion. Discussion of major findings is lacking in general. The conclusion section is weak and can be improved.

Response: We have edited the discussion and conclusion section as advised.

**R1 Comment 9:** Academic writing:

1. Avoid use of unnecessary capitalizations

Response: This has been checked and edited throughout.

2. Use past tense to report methods and results

Response: This has been checked and edited throughout.

3. Use hyphenation where necessary (e.g.: face-to-face)

Response: This has been checked and edited throughout

4. Inconsistent use of percentage (example: 53%, 27. 2%, 56.9% etc.)

Response: This has been checked and edited throughout

5. Inconsistent use of the Oxford comma before “and”.

Response: This has been checked and edited throughout

6. Commas missing in many sentences.

Response: This has been checked and edited throughout

7. Check spellings

Response: This has been checked and edited throughout

8. Inconsistent use of the words “aging” and “ageing”; “elderly” and “older people”. Use the same term throughout the text.

Response: This has been checked and edited throughout

9. Recheck the text. The flow of paragraphs can be improved. Hope the suggestions are useful.

Best wishes.

Response: This has been checked and edited throughout. Thank you so much.

**Reviewer #2:**

Thank you for the constructive comments.

**R2 Comment 1:** The topic is interesting and explores an important area of public health research. The article is good. However, there are some changes that need to made to make the article even better.

Response: Thank you so much. We have revised this manuscript considering all the comments.

**R2 Comment 2:** Overall Suggestions: There are minor errors in grammar/language. Please kindly proofread the article.

Response: This has been checked and edited throughout

**R2 Comment 3:** o Also some rearranging and adding of text may be necessary.

Response: This has been checked and edited throughout

**R2 Comment 4:** o Please make sure that the numbers in tables are correct. Details are in my comments below.

Response: This has been checked and edited throughout

**R2 Comment 5:** Title page:

o Line 10: Please use the correct spelling of ‘Hetauda’

Response: We have corrected it now

**R2 Comment 6:** Abstract:

o Line 50: Keywords not in alphabetical order

Response: We have now kept keywords in alphabetical order.

**R2 Comment 7:** Materials and Methods:

o Line 86: What do you mean by structured interview? You have mentioned that the questionnaire itself is semi-structured. Do you mean face to face interview?

Response: Yes, we meant face to face interview with semi-strucured questionnaire. We have corrected that.

**R2 Comment 8:**

o Line 102: Please explain how this was a stratified proportionate random sampling? Did you have a sampling frame? If yes, please mention it.

Response: The sampling frame was elderly people residing two sub-metropolitan cities. Eight wards from each sub-metropolitan selected randomly were strata in this study. The population of wards were identified from administrative office and number of participants from each ward was calculated proportionately.

We have mentioned it in line 100 – 105.

**R2 Comment 8:** o Line 134: The term elderly is defined here. As this study is about elderly people, it should be moved it up in the text so that reader can understand the context

Response: I have moved the text above in the introduction part and it is now in line 67 – 68.

We have ensured this in the revision.

**R2 Comment 9:** o Line 165: Please specify which version of SPSS was used

Response: We have specified the version of SPSS used. I used SPSS version 16. It is stated in line 160 -161.

**R2 Comment 10:** o 167-169: Where are the results of multivariate analysis? Where is the Chi-square test used?

Response: We have added the tables with the results for chi-square test and multivariate analysis. Table 4 shows the bivariate analysis and table 5 and 6 states the finding of multivariable analysis.

**R2 Comment 11:** Results

o Line 182: In Table 1: for Age distribution, the percentage does not sum up to 100.0%. Please correct it.

Response: This has been checked and corrected. Thank you.

**R2 Comment 12:** o Line 184-186: Not clear what the author wants to say and may be confusing for the readers. Please re-write to bring clarity.

Response: We have edited this to two categories, with family or by themselves.

**R2 Comment 13:** o Line 200-203: How did you assess the blood pressure at the time of visit? Did you measure the blood pressure yourselves? It is not mentioned in the methodology or tools used.

Response: As a qualified physician, the PI measured the blood pressure. This is now mentioned in methodology and is in line 130 – 134.

**R2 Comment 14:** o Line 209: Measurement of proteinuria by the researcher and the tools used for this process is not mentioned in the methodology.

Response: Based on this revision after adding and rearranging the findings to match the objectives, we have removed proteinuria from results hence not mentioned it in methods.

**R2 Comment 15:** What you are trying to present as health needs is not clearly depicted in the results and discussion section.

Response: We have revised the title as ‘ Morbidities, health problems, health care seeking and utilization behaviour among elderly residing on urban areas of eastern Nepal: a cross-sectional study’ to improve the focus and enhance clarity. We have ensured this is reflected in the objectives as well as presented in a similar manner in results and discussion,

**R2 Comment 16:** References: Some of the references are not according to the journal guidelines. Please make required changes.

Response: We have made these changes in the references.

Thank you ever so much for all the comments.
