# Supplementary material for: Morbidities, health problems, health care seeking and utilization behaviour among elderly residing on urban areas of eastern Nepal: A cross-sectional study
Source: PLoS One. 2022 Sep 7;17(9):e0273101. doi: 10.1371/journal.pone.0273101 (PMC9451091; doi:10.1371/journal.pone.0273101)
Supplement: Attachment — Submitted filename: Response to reviewers.docx [file pone.0273101.s004.docx]

Dear reviewer,

I kindly appreciate all your constructive comments and effort in making this manuscript better. I have tried my best to respond to all your comments.

Reviewer 2

Thank you for the appreciation.

Reviewer 3

Thank you for the constructive comments.

**R3 Comment 1**: The article is very well written and the enormous effort undertaken during the study design, data analysis and writing of the manuscript is greatly appreciated. Indeed, the issues of elderly is an important issue and in developing countries like Nepal, more such research and articles are appreciated.
Response: Thank you for the appreciation. We will continuously look forward to explore the issues of elderly and are motivated to conduct research in this area

**R3 Comment 2**: Line 26 (abstract) inconsistent use of hyphenation in ‘health care seeking’

Response: I have removed the hyphenation and the word now reads as health care seeking behaviour in line 26.

**R3 Comment 3:** Line 216 ‘6’ to be written as six

Response: This has been edited and number is written in text in line 221.

**R3 Comment 4:** Line 276 comma missing.

Response: This has been edited.

**R3 Comment 5:** Yes, the manuscript is well written. However, the data shared is coded and codes for limited variables are given so reviewer is unable to decide with full confidence. Eg GD column in data sheet reads as ‘multimorbidity’ which has 271 rows coded as 1 (51.13%). Reviewer cannot be sure what this implies. Most of the conclusions seem possible when the questionnaire is reviewed.
Response: Thank you. We shared a basic excel sheet and a SPSS data set. The excel sheet being a basic data set we tried various outcomes. The multimorbidity stated in that data set includes the relative health problem also. But later for the analysis the morbidity and multimorbidity was classified based upon preexisting health condition. The SPSS data set has been recoded again for analysis and it gives more clarity on data analysis.

**R3 Comment 6:** Line 99 Sampling method has been explained and eight wards have been selected as per the manuscript but data sheet shows missing data in 45 participants and there are 9 wards in one of the cities i.e. Dharan (if the number allotted after Dharan is the ward no in the data sheet). More clarity would be there if authors showed the number in sampling frame and ward wise population as reviewer believes this is a great strength of this article.
Response: The ward wise population has been cited accordingly in the methodology section in line 104. The data representing ward number has been revised and corrected accordingly.

**R3 Comment 7:** Line 112-116 Calculation of sample size: The formula used one proportion sample size seems fine, but in the stated formula, I am not sure where the power (85%) is used. The rationale for choosing prevalence of unmet health care needs in people suffering with ‘hypertension’ along with the full calculation would clarify readers.
Response: The calculation of sample size is elaborated accordingly in line 112-118. The rationale of choosing prevalence of unmet health care need is now stated which reads as “This prevalence suggests the scenario of health care seeking practice with one of the common morbidity among elderly” in line 114-115.

**R3 Comment 8:** Line 118 onwards in “variables studied’’ there is inconsistent writing style with some of the variables being described in paragraph and some with heading.
Response: This has been edited and variables are now described in paragraph.

**R3 Comment 9:** Line 119 The questionnaire is ‘pre-tested’. Elaborating the pre-testing technique would strengthen the article as the questionnaire shared is in great detail with translation to local language. This work is not reflected in the manuscript.

Response: The pretesting was done among the elderly visiting the BP Koirala Institute of Health Sciences. We have added about pretesting in the manuscript as “A questionnaire intended to fulfil the study objective was developed based on different studies among the elderly. The questions were discussed among authors for validity. The translation of questions into the local language was done. Elderly visiting the BP Koirala Institute of Health Sciences were interviewed. Authors reviewed the acquired answers with an amendment to the questionnaire.” In line 122-126.

**R3 Comment 10:** Line 134-136 BDI II scale used for depression but the result of this has not been expressed in ‘Results’ section. If authors are not publishing this, maybe they can omit from methods section too. It also raises a question why only ‘depression’ was assessed and not other mental health issues.

Response: It has been presented in result section as psychological problem. The rationale of assessing this mental health problem is now included and it reads as “Depression is one of the most common psychological problem in elderly population” in line 142-143.

**R3 Comment 11:** Line 140-141 ICPC is used for health problems which is a standard in itself so the sentence following it with Ref 30 seems only to add to the authors long list of references (study among inmates vs study of elderly)
Response: By citing the paper on inmates, our intentions were to show that ICPC has been used for research in Nepal. However to avoid confusion the reference has been revised and removed in line 148.

**R3 Comment 12:** Line 149 Reference for this variable (if provided) would be good.
Response: The reference for tobacco users has now been provided to that variable in line 156.

**R3 Comment 13:** Table 3 Mentions “Pention camp’’, the terminology might not be clear to many people so elaboration below table on what kind of ‘health facility’ would add to clarity.
Response: The footnote in the table now describes in a brief about the pension camp which reads as “Pension camp is the common term used by people for pension paying office for the Ex Gurkha army personnel, which also have a health facility with medical personnel and doctors which provides health care service free of cost” in line 216 – 218.

**R3 Comment 13:** In ‘Last visit to any health facility’, one of the options reads as ‘Never’ with 47 responders choosing it. Does it mean in their 60 years, they never visited a health facility?
Response: Yes, it was the response from those 47 participants. The added response of these participant was taking home remedies and seeking traditional healer.


**R3 Comment 14:** Discussion Section: Overall slightly weak. Authors have compared their findings with other researches but they have not explained why the variations are there. Eg for HTN, eye (66.2 vs 19.1), dental issues etc
Response: We have revised the discussion and added explanation for such variations in line 312 -315.

**R3 Comment 15:** Line 257 The article including the title of the article refers the study setting as urban eastern Nepal, while in this line the article claims to represent whole Nepal in terms of culture, ethnicity, religion, education, socioeconomic status, lifestyle which seems contradictory in itself.
Response: We have revised it and edited as “represent urban areas of the country” in line 264.

**R3 Comment 16:** Flow of article in chronic morbidities section needs to be worked out. Eg

- Line 271-275 Manuscript is describing articles
- Line 276-277 Manuscript gives a broad comment
- Line 277 onwards manuscript starts to describe individual articles
- When discussing, it is better to discuss one variable and then start others, broad statements are better at beginning of discussion to start a variable discussion or the end.

Response: We have revised the discussion and edited as per the comments in line 273-301.

**R3 Comment 17:** Line 277 The prevalence of HTN is 34% in this study. The manuscript reports a ‘similar’ range of 27-57% in other studies. Maybe the authors should reconsider the term ‘similar’ considering the expressed range.

Response: This has been revised and edited. Similarly has been removed and it has been stated as “Other studies have reported a prevalence of hypertension ranging from 27-57% among the elderly” in line 293 - 294.

**R3 Comment 18:** References in discussion section is missing. Eg Line 280, Line 291, Line 303. It gives more clarity to readers if reference is given immediately after a particular article is discussed and not at the end of the paragraph. Author is advised to check this throughout the discussion section.
Response: We have revised this and edited accordingly. The references is now provided after each statement.

**R3 Comment 19:** References:
One Ref 37 is of the year 2022 while most others are relatively not recent so maybe the author can add more recent articles in discussion section while revising the manuscript. Author has 56 references; some references might need reconsideration for citation as stated above.

Response: This has been revised and we have tried to include the recent articles in discussion section.
